# Associations of Fetal and Infant Growth Patterns With Early Markers of Arterial Health in School-Aged Children

**DOI:** 10.1001/jamanetworkopen.2022.19225

**Published:** 2022-06-29

**Authors:** Romy Gonçalves, Clarissa J. Wiertsema, Carolina C. V. Silva, Giulietta S. Monasso, Romy Gaillard, Eric A. P. Steegers, Susana Santos, Vincent W. V. Jaddoe

**Affiliations:** 1 The Generation R Study Group, Erasmus MC, University Medical Center, Rotterdam, the Netherlands; 2 Department of Pediatrics, Sophia’s Children’s Hospital, Erasmus MC, University Medical Center, Rotterdam, the Netherlands; 3 Department of Obstetrics and Gynaecology, Sophia’s Children’s Hospital, Erasmus MC, University Medical Center, Rotterdam, the Netherlands

## Abstract

**Importance:**

Fetal life and infancy might be critical periods for predisposing individuals to develop cardiovascular disease in adulthood.

**Objective:**

To examine the associations of fetal and infant weight growth patterns with early markers of arterial health.

**Design, Setting, and Participants:**

This population-based prospective cohort study was conducted from early fetal life onward among 4484 offspring of women in Rotterdam, the Netherlands, delivering between April 1, 2002, and January 31, 2006. Statistical analysis was performed between January 1 and August 31, 2021.

**Exposures:**

Estimated fetal weight was measured in the second and third trimester. Data on weight and gestational age at birth were collected from midwives. Infant weight was measured at 6, 12, and 24 months.

**Main Outcomes and Measures:**

The common carotid intima-media thickness (cIMT) and carotid distensibility were measured as early markers of arterial health.

**Results:**

Follow-up measurements were available for 4484 children (2260 girls [50.4%]; median age, 9.7 years [95% range, 9.3-10.5 years]; and 2578 [57.5%] of Dutch ethnicity). Gestational age at birth was not associated with markers of arterial health. A 500-g–higher birth weight was associated with increased cIMT (standard deviation score [SDS], 0.08 mm [95% CI, 0.05-0.10 mm]) and a lower carotid distensibility (SDS, −0.05 × 10^–3^ kPa^–1^; [95% CI, −0.08 to −0.03 × 10^–3^ kPa^–1^]). Compared with children with a birth weight of 2500 to 4500 g, those weighing more than 4500 g had the lowest carotid distensibility (difference in SDS, −0.22 × 10^–3^ kPa^–1^ [95% CI, −0.42 to −0.02 × 10^–3^ kPa^–1^]). Conditional regression analyses showed that higher third-trimester fetal weight and birth weight were associated with increased cIMT (difference in SDS: third-trimester fetal weight, 0.08 mm [95% CI, 0.04-0.12 mm]; birth weight, 0.05 mm [95% CI, 0.01-0.09 mm]) and that higher weight at 6, 12, and 24 months was associated with increased cIMT (difference in SDS: 6 months, 0.05 mm [95% CI, 0.01-0.10 mm]; 12 months, 0.06 mm [95% CI, 0.02-0.10 mm]; and 24 months, 0.07 mm [95% CI, 0.03-0.11 mm]) and lower carotid distensibility (difference in SDS: 6 months, –0.04 × 10^–3^ kPa^–1^ [95% CI, –0.09 to –0.001 × 10^–3^ kPa^–1^]; 12 months, –0.05 × 10^–3^ kPa^–1^ [95% CI, –0.09 to –0.01 × 10^–3^ kPa^–1^]; and 24 months, –0.10 × 10^–3^ kPa^–1^ [95% CI, –0.15 to –0.06 × 10^–3^ kPa^–1^]). Compared with children with normal fetal and infant growth, children with normal fetal growth that was followed by accelerated infant growth had the highest cIMT (SDS, 0.19 mm [95% CI, 0.07-0.31 mm]) and lowest carotid distensibility (SDS, −0.16 × 10^–3^ kPa^–1^ [95% CI, −0.28 to −0.03 × 10^–3^ kPa^–1^]). The observed associations were largely explained by childhood body mass index.

**Conclusions and Relevance:**

In this cohort study of 4484 children aged approximately 10 years, higher fetal and infant weight growth patterns were associated with early markers of impaired arterial health. Childhood body mass index seemed to be involved in the underlying pathways of the observed associations.

## Key Points

**Question:**

How are fetal and infant growth patterns associated with early markers of arterial health in children aged 10 years?

**Findings:**

In this population-based cohort study of 4484 children, those with normal fetal growth that was followed by accelerated infant growth had the highest carotid intima-media thickness and the lowest carotid distensibility. Childhood body mass index seemed to be involved in the pathways underlying the observed associations.

**Meaning:**

This study suggests that both higher fetal and infant weight growth patterns are associated with early markers of impaired arterial health.

## Teaser

This cohort study examines the associations of fetal and infant weight growth patterns with early markers of arterial health among school-aged children.

## Introduction

Children with low birth weight or high birth weight and then subsequent high infant growth seem to be at risk for cardiovascular disease in adulthood.^1,2^ Study findings suggest that variation in growth during both fetal life and infancy is associated with an adverse distribution of body fat and an adverse cardiovascular risk profile from school age onward.^3,4,5^ These findings suggest that altered growth in early life might predispose individuals to develop atherosclerosis and subsequent cardiovascular disease in adulthood.^6^ Results from postmortem pathologic studies show atherosclerosis of large arteries in fetuses, children, and adolescents.^7,8,9^ Studies also show that an adverse fetal environment is associated with noninvasive markers of arterial health and atherosclerosis, such as carotid intima-media thickness (cIMT) and carotid distensibility, in adolescence.^10,11^ In a recent systematic review assessing risk factors in the first 1000 days of life, a consistent association was reported between small size for gestational age (SGA) at birth with increased cIMT in individuals aged 18 years or younger.^10^ However, another study reported a positive association of higher birth weight with cIMT, independent of childhood obesity.^12^ These findings suggest a nonlinear association of birth weight with childhood cIMT. Identification of critical periods in fetal life and infancy associated with arterial health and atherosclerosis might contribute to novel prevention strategies.^13,14,15,16,17,18^

In a population-based prospective cohort study of 4484 children, we examined the associations of fetal and infant weight growth patterns with cIMT and carotid distensibility at 10 years of age. We were specifically interested in the identification of critical periods and combinations of fetal and infant weight growth patterns.

## Methods

### Study Population

This study was embedded in the Generation R Study, a population-based prospective cohort study from early fetal life onward.^19^ Pregnant women with a delivery date between April 1, 2002, and January 31, 2006, living in Rotterdam, the Netherlands, were eligible to participate. Details on response and follow-up have been described previously.^19^ Information on fetal or infant growth was available for 8625 singleton births. Analyses were restricted to a subgroup of 4484 children for whom we had information on early markers of arterial health. The flowchart of participants is given in eFigure 1 in the Supplement. Written informed consent was provided by the parents for all children. The medical ethics committee of Erasmus Medical Center approved the study. This study followed the Strengthening the Reporting of Observational Studies in Epidemiology (STROBE) reporting guideline.^20^

### Fetal and Infant Growth Measures

As previously described,^19^ ultrasonography examinations were performed in the first trimester (median, 12.4 weeks; range, 10.1-13.9 weeks), second trimester (median, 20.5 weeks; range, 18.0-25.0 weeks), and third trimester (median, 30.4 weeks; range, 25.3-39.2 weeks). We measured second- and third-trimester fetal head circumference, abdominal circumference, and femur length by using standardized ultrasonography procedures.^19,21^ We used the formula of Hadlock et al^22^ to calculate gestational age–adjusted estimated fetal weight and then the calculated standard deviation score (SDS).^23^ We constructed the gestational age–adjusted SDS for the first-trimester fetal crown-to-rump length in a subgroup of mothers.^23^ A detailed description of this measurement can be found in the eMethods in the Supplement.

Sex, gestational age at birth, and birth weight were collected from midwives. In our study population, birth weight was standardized based on gestational age and sex according to growth charts by Niklasson et al.^24,25^ These growth charts are well known and commonly used growth charts for northern European countries, including the Netherlands. For the analyses, we used internal percentiles for comparison. We used the clinical cutoffs for gestational age and weight at birth. Gestational age was categorized as preterm (<37 weeks), term (37-42 weeks), and postterm (>42 weeks). Birth weight was categorized as low (<2500 g), normal (2500-4500 g), and high (>4500 g). Children born SGA were defined as those with a gestational age–adjusted and sex-adjusted SDS for birth weight below the 10th percentile, and those born large size for gestational age (LGA) were defined as those with a gestational age–adjusted and sex-adjusted SDS for birth weight above the 90th percentile.

Infant weight was measured at approximately 6 months of age (median, 6.2 months; 95% range, 5.2-8.3 months), 12 months (median, 11.1 months; 95% range, 10.1-12.5 months), and 24 months (median, 24.8 months; 95% range, 23.4-28.2 months).^19^ We created age-adjusted and sex-adjusted SDSs using Dutch reference growth charts in Growth Analyser, version 4.0 (Growth Analyser BV).^19^

We prospectively constructed 9 categories of fetal and infant weight change variables. Fetal weight change was defined as an increase in the SDS between the second trimester and birth. Infant weight change was defined as an increase in the SDS from birth to 24 months (2929 of 3651 children). If weight at 24 months was not available, we used weight at 11 months (587 of 3651 children), and if weight at 11 months was not available, we used weight at 6 months (135 of 3651 children). We considered an increase of more than 0.67 SD between time points as growth acceleration and a decrease of more than 0.67 SD between time points as growth deceleration, reflecting the difference between 2 percentile lines on the growth charts.

### Childhood Common cIMT and Distensibility

At the median age of 9.7 years (95% range, 9.3-10.5 years), we measured cIMT and carotid distensibility using the Logiq E9 device (GE Medical Systems) and obtained 6 recordings. Carotid intima-media thickness was computed at the “far wall” as the mean distance between lumen-intima and media-adventitia borders. Distensibility was defined as the relative change in lumen area during systole for a given pressure change. Children with at least 1 successful cIMT or distensibility measurement were included. The overall mean cIMT and carotid distensibility were the main outcomes of interest. For the final analyses, distensibility was log transformed to deal with a skewed distribution. We constructed SDSs ([observed value − mean] / SD) for the childhood outcome measures to enable comparison of effect estimates. A more detailed description of the measurement is given in the eMethods in the Supplement.

### Covariates

Information on maternal age, prepregnancy weight, parity, race and ethnicity (Cape Verdean, Dutch, Dutch Antilles, Moroccan, Surinamese, Turkish, and other [which included African; North, Central, and South American non-Western; North, Central, and South American Western; Asian non-Western; Asian Western; European; Indonesian; and Oceanian]), educational level, smoking, folic acid supplementation, and gestational hypertensive disorders was obtained by questionnaires and registries.^19^ Maternal height was measured and prepregnancy body mass index (BMI; calculated as weight in kilograms divided by height in meters squared) was calculated. At a median age of 9.7 years (95% range, 9.3-10.5 years), child height and weight were measured. We calculated the sex-adjusted and gestational age–adjusted BMI SDS. Race and ethnicity were included as covariates because of their association with both fetal and infant growth as with carotid measurements. The roles of the covariates of interest are presented in a directed acyclic graph (eFigure 2 in the Supplement).

### Statistical Analysis

Statistical analysis was performed between January 1 and August 31, 2021. First, we described maternal, fetal, and childhood characteristics. We performed a nonresponse analysis by comparing characteristics of children with and without outcome assessments by using the independent *t* test, the Mann-Whitney test, and the χ^2^ test. The nonlinearity of the associations was assessed by checking linear assumptions and then was ruled out. Second, we assessed the associations of gestational age at birth, birth weight, and size for gestational age at birth with cIMT and carotid distensibility at 10 years of age using linear regression models. Third, we additionally explored the associations of weight measurements at different fetal and infant ages starting from the first trimester of pregnancy with carotid measurements using linear regression models. First-trimester growth was available for a small subgroup (n = 941). To identify independent critical weight periods, we performed conditional regression analysis that took into account the correlations between the weight measurements.^4,26^ We constructed weight variables statistically independent from weight measurements at earlier time points by using standardized residuals resulting from linear regression models of weight from all prior weight measurements.^27^ This approach allows for the simultaneous inclusion of all weight measurements in 1 linear regression model to identify critical periods of growth using childhood carotid measurements. Data for weight at all time points was required for these analyses. The first-trimester measurements were excluded owing to the limited sample size.

Fourth, we categorized fetal (second trimester to birth) and infant (birth to 24 months) weight change into 3 groups (growth deceleration, normal growth, and growth acceleration) and created a combined variable that reflected 9 different growth patterns. We used multivariable linear regression models to explore associations of fetal and infant weight changes combined with carotid measurements. We performed a sensitivity analysis restricting the study population to children with weight measurements available at 24 months (n = 2789). For all analyses, the basic models were adjusted for child’s sex and age at outcome measurement. The confounder model, which we considered the main model, was additionally adjusted for maternal age, prepregnancy BMI, educational level, race and ethnicity, folic acid use, smoking, and gestational hypertensive disorders. The mediator model additionally included childhood BMI. Potential confounders were identified based on previous literature, and we selected those that fulfilled the graphical criteria for confounding in a directed acyclic graph and changed the effect estimates more than 10% after addition to the crude model. We tested for the statistical interaction of sex and race and ethnicity, but no statistically significant interactions were observed. As exposures were correlated, we did not correct for multiple testing and present significance levels at both *P* < .05 and *P* < .001. Missing data in covariates (range, 0%-23%) were multiply imputed using the Markov Chain Monte Carlo method. Ten imputed data sets were created and analyzed together.^28^ Statistical analyses were performed using SPSS, version 25.0 for Windows (SPSS Inc).

## Results

### Characteristics of Participants

Table 1 shows the characteristics of the participants from imputed data. Follow-up measurements were available for 4484 children (2260 girls [50.4%]; median age, 9.7 years [95% range, 9.3-10.5 years]; and 2578 [57.5%] of Dutch ethnicity). eTable 1 in the Supplement shows the characteristics of the participants from observed data. eTable 2 in the Supplement shows that, compared with the study population, mothers of children without outcome measurements more often had a lower educational level (2854 of 4141 [68.9%] vs 2297 of 4484 [51.2%]; *P* < .001), were more often of non-European descent (2205 of 4141 [53.2%] vs 1576 of 4484 [35.1%]; *P* < .001), and smoked more often during pregnancy (1273 of 4141 [30.7%] vs 1081 of 4484 [24.1%]; *P* < .001) and also used folic acid supplementation less often during pregnancy (2504 of 4141 [60.5%] vs 3412 of 4484 [76.1%]; *P* < .001). Children not participating in the study were slightly more often born preterm than those participating in the study (250 of 4141 [6.0%] vs 207 of 4484 [4.6%]; *P* = .01) and had lower third-trimester estimated fetal weight (median weight, 1580 g [95% range, 1141-2200 g] vs 1605 g [95% range, 1179-2218 g]).

**Table 1.** Characteristics of the Study Population^a^

Characteristic	Participants, No. (%) (N = 4484)
**Maternal**	
Age at enrollment, median (95% range), y	31.2 (20.3-39.6)
Prepregnancy BMI, median (95% range)	22.7 (17.8-34.1)
Nulliparous	2628 (58.6)
Educational level, higher education	2187 (48.8)
Race and ethnicity	
Cape Verdean	184 (4.1)
Dutch	2578 (57.5)
Dutch Antilles	99 (2.2)
Moroccan	211 (4.7)
Surinamese	345 (7.7)
Turkish	318 (7.1)
Other^b^	749 (16.7)
Continued smoking during pregnancy	1081 (24.1)
Did not use folic acid supplement	1072 (23.9)
**Fetal**	
First trimester	
Gestational age, median (95% range), wk	12.4 (10.6-13.8)
Crown-to-rump length, mean (SD), mm	61.0 (11.6)
Second trimester	
Gestational age, median (95% range), wk	20.5 (18.6-23.3)
Estimated fetal weight, median (95% range), g	364 (246-624)
Third trimester	
Gestational age, median (95% range), wk	30.4 (28.5-33.0)
Estimated fetal weight, median (95% range), g	1605 (1179-2218)
**Birth**	
Child sex, female	2260 (50.4)
Gestational age at birth, median (95% range), wk	40.1 (35.9-42.3)
<37	207 (4.6)
37-42	4060 (90.5)
>42	217 (4.8)
Birth weight, median (95% range), g	3450 (2255-4485)
<2500	196 (4.4)
2500-4500	4182 (93.4)
>4500	101 (2.3)
Sex- and gestational age–adjusted birth weight	
Small (<10th percentile)	447 (10.0)
Appropriate (10th-90th percentile)	3582 (80.0)
Large (>90th percentile)	447 (10.0)
**Infant, median (95% range)**	
At 6-mo visit	
Age at visit, mo	6.2 (5.2-8.3)
Weight, kg	7.8 (6.2-9.8)
At 12-mo visit	
Age at visit, y	11.1 (10.1-12.5)
Weight, kg	9.6 (7.7-11.8)
At 24-mo visit	
Age at visit, y	24.8 (23.4-28.2)
Weight, kg	12.8 (10.3-16.2)
**Childhood**	
Age at follow-up, median (95% range), y	9.7 (9.3-10.5)
BMI, median (95% range)	17.0 (14.0-24.9)
Carotid intima-media thickness, mean (SD), mm	0.46 (0.04)
Carotid distensibility, median (95% range), ×10^–3^ kPa^–1^	55.9 (37.1-85.5)

Abbreviation: BMI, body mass index (calculated as weight in kilograms divided by height in meters squared).

^a^ Characteristics are based on the pooled results after multiple imputations.

^b^ Included African; North, Central, and South American non-Western; North, Central, and South American Western; Asian non-Western; Asian Western; European; Indonesian; and Oceanian.

### Birth Outcomes

Table 2 shows the associations of birth outcomes with childhood carotid measurements adjusted for confounders and childhood BMI (basic models are shown in eTable 3 in the Supplement). Gestational age was not associated with childhood carotid measurements. An increase in birth weight of 500 g was associated with increased cIMT (SDS, 0.08 mm [95% CI, 0.05-0.10 mm]) and a lower carotid distensibility (SDS, −0.05 × 10^–3^ kPa^–1^ [95% CI, −0.08 to −0.03 × 10^–3^ kPa^–1^]) (Table 2). Compared with children with a birth weight of 2500 to 4500 g, those with a birth weight of more than 4500 g had the lowest carotid distensibility (difference in SDS, −0.22 × 10^–3^ kPa^–1^ [95% CI, −0.42 to −0.02 × 10^–3^ kPa^–1^]). Similarly, a 1-SDS increase in gestational age–adjusted birth weight was associated with increased cIMT (SDS, 0.08 mm [95% CI, 0.05-0.11 mm]) and a lower carotid distensibility (SDS, −0.07 × 10^–3^ kPa^–1^ [95% CI, −0.10 to −0.04 × 10^–3^ kPa^–1^]). Being SGA was associated with decreased cIMT (SDS, −0.14 mm [95% CI, −0.24 to −0.04 mm]) and a higher carotid distensibility (SDS, 0.12 × 10^–3^ kPa^–1^ [95% CI, 0.02-0.22 × 10^–3^ kPa^–1^]). The associations of birth weight greater than 4500 g, SGA, and LGA with lowered carotid distensibility were attenuated into nonsignificance after additional adjustment for childhood BMI.

**Table 2.** Associations of Birth Outcomes With Childhood Carotid Measurements

Birth outcome	No.	Difference in SDS (95% CI)^a^			
		Carotid intima-media thickness, mm (N = 4484)		Carotid distensibility, ×10^–3^ kPa^–1^ (n = 4304)	
		Confounder model	BMI model	Confounder model	BMI model
Birth weight, g					
<2500	196	−0.06 (−0.20 to 0.09)	−0.05 (−0.20 to 0.09)	0.11 (−0.04 to 0.25)	0.10 (−0.05 to 0.24)
2500-4500	4182	[Reference]	[Reference]	[Reference]	[Reference]
>4500	101	0.15 (−0.05 to 0.35)	0.14 (−0.06 to 0.33)	−0.22 (−0.42 to −0.02)^b^	−0.18 (−0.37 to 0.02)
Continuously (per 500 g)	4479	0.08 (0.05 to 0.10)^c^	0.07 (0.04 to 0.10)^c^	−0.05 (−0.08 to −0.03)^c^	−0.04 (−0.07 to −0.01)^b^
Size for gestational age at birth					
Small (<10th percentile)	447	−0.14 (−0.24 to −0.04)^b^	−0.13 (−0.23 to −0.03)^b^	0.12 (0.02 to 0.22)^b^	0.10 (−0.004 to 0.20)
Appropriate (10th-90th percentile)	3582	[Reference]	[Reference]	[Reference]	[Reference]
Large (>90th percentile)	447	0.11 (0.12 to 0.21)^b^	0.10 (0.001 to 0.20)^b^	−0.10 (−0.20 to −0.001)^b^	−0.07 (−0.17 to 0.03)
Continuously (per 1-SD g)	4476	0.08 (0.05 to 0.11)^c^	0.07 (0.04 to 0.10)^c^	−0.07 (−0.10 to −0.04)^c^	−0.05 (−0.08 to −0.02)^b^
Gestational age at birth, wk					
<37	207	−0.08 (−0.22 to 0.06)	−0.08 (−0.22 to 0.06)	0.06 (−0.08 to 0.20)	0.06 (−0.08 to 0.20)
37-42	4060	[Reference]	[Reference]	[Reference]	[Reference]
>42	217	0.07 (−0.07 to 0.21)	0.07 (−0.07 to 0.21)	0.03 (−0.12 to 0.17)	0.03 (−0.11 to 0.17)
Continuously (per week)	4484	0.02 (−0.001 to 0.03)	0.02 (−0.001 to 0.03)	−0.01 (−0.02 to 0.01)	−0.01 (−0.02 to 0.01)

Abbreviations: BMI, body mass index (calculated as weight in kilograms divided by height in meters squared); SDS, standard deviation score.

^a^ Values are regression coefficients (95% CIs) that were obtained from multivariable linear regression models and reflect the differences in carotid intima-media thickness (SDS) and carotid distensibility (SDS) for birth outcomes. Estimates are from multiple imputed data. The confounder model is adjusted for child age at the outcome visit and sex, maternal age, prepregnancy BMI, educational level, race and ethnicity, folic acid use, smoking, and gestational hypertensive disorders. The BMI model is the confounder model additionally adjusted for sex-adjusted and gestational age–adjusted child BMI at the outcome measurement.

^b^ *P* < .05.

^c^ *P* < .001.

### Critical Fetal and Infant Periods

Results from conditional regression analyses showed that higher third-trimester fetal weight, birth weight, and weight at 6, 12, and 24 months were all independently associated with increased cIMT (difference in SDS: third-trimester fetal weight, 0.08 mm [95% CI, 0.04-0.12 mm]; birth weight, 0.05 mm [95% CI, 0.01-0.09 mm]; 6 months, 0.05 mm [95% CI, 0.01-0.10 mm]; 12 months, 0.06 mm [95% CI, 0.02-0.10 mm]; and 24 months, 0.07 mm [95% CI, 0.03-0.11 mm]), whereas higher weight at 6, 12, and 24 months were all independently associated with lower carotid distensibility (difference in SDS: 6 months, –0.04 × 10^–3^ kPa^–1^ [95% CI, –0.09 to –0.001 × 10^–3^ kPa^–1^]; 12 months, –0.05 × 10^–3^ kPa^–1^ [95% CI, –0.09 to –0.01 × 10^–3^ kPa^–1^]; and 24 months, –0.10 × 10^–3^ kPa^–1^ [95% CI, –0.15 to –0.06 × 10^–3^ kPa^–1^]) (Table 3). After additional adjustment for childhood BMI, only a higher infant weight at 24 months remained associated with lower carotid distensibility (SDS, −0.08 × 10^–3^ kPa^–1^ [95% CI, −0.12 to −0.03 × 10^–3^ kPa^–1^]). The basic models are given in eTable 4 in the Supplement. eTable 5 in the Supplement shows that no associations with markers of arterial health were observed for first-trimester length and fetal weight at 20 weeks of gestation.

**Table 3.** Associations of Fetal and Infant Growth With Childhood Carotid Measurements From Conditional Analyses

Infant and fetal weight	Difference in SDS (95% CI)^a^			
	Carotid intima-media thickness, mm (n = 2249)		Carotid distensibility, ×10^–3^ kPa^–1^ (n = 2137)	
	Confounder model	BMI model	Confounder model	BMI model
At fetal weight 20 wk	0.04 (−0.01 to 0.08)	0.03 (−0.01 to 0.08)	−0.01 (−0.05 to 0.04)	−0.00 (−0.05 to 0.04)
At fetal weight 30 wk	0.08 (0.04 to 0.12)^b^	0.08 (0.04 to 0.12)^b^	−0.03 (−0.08 to 0.01)	−0.03 (−0.07 to 0.02)
At birth	0.05 (0.01 to 0.09)^c^	0.05 (0.004 to 0.09)^c^	−0.01 (−0.05 to 0.04)	0.01 (−0.04 to 0.05)
At 6 mo	0.05 (0.01 to 0.10)^c^	0.05 (0.004 to 0.09)^c^	−0.04 (−0.09 to −0.001)^c^	−0.01 (−0.06 to 0.03)
At 12 mo	0.06 (0.02 to 0.10)^c^	0.05 (0.01 to 0.09)^c^	−0.05 (−0.09 to −0.01)^c^	−0.03 (−0.07 to 0.02)
At 24 mo	0.07 (0.03 to 0.11)^c^	0.06 (0.02 to 0.10)^c^	−0.10 (−0.15 to −0.06)^b^	−0.08 (−0.12 to −0.03)^b^

Abbreviations: BMI, body mass index (calculated as weight in kilograms divided by height in meters squared); SDS, standard deviation score.

^a^ Values are regression coefficients (95% CIs) and reflect the differences in carotid intima-media thickness (SDS) and carotid distensibility (SDS) per SDS change in infant and fetal weight from conditional models. Estimates are from multiple imputed data. The confounder model is adjusted for child age at the outcome visit and sex, maternal age, prepregnancy BMI, educational level, race and ethnicity, folic acid use, smoking, and gestational hypertensive disorders. The BMI model is the confounder model additionally adjusted for sex-adjusted and gestational age–adjusted child BMI at the outcome measurement.

^b^ *P* < .001.

^c^ *P* < .05.

### Fetal and Infant Growth Patterns

Table 4 shows that, compared with children with normal fetal and infant growth, those with normal fetal growth that was followed by accelerated infant growth had the highest cIMT (SDS, 0.19 mm [95% CI, 0.07-0.31 mm]) and the lowest carotid distensibility (SDS, −0.16 × 10^–3^ kPa^–1^ [95% CI, −0.28 to −0.03 × 10^–3^ kPa^–1]^). The association of normal fetal growth that was followed by accelerated infant growth and carotid distensibility was attenuated into nonsignificance after additional adjustment for childhood BMI. No other consistent associations were observed. The corresponding basic models are shown in eTable 6 in the Supplement. eTable 7 in the Supplement shows maternal prepregnancy BMI for the different fetal and infant weight growth patterns. The results were largely similar when we restricted the analyses for the associations of fetal and infant growth patterns with arterial health markers to children with weight measurements at 24 months (eTable 8 in the Supplement).

**Table 4.** Associations of Fetal and Infant Growth Patterns With Childhood Carotid Measurements

Fetal and infant growth pattern	No.	Difference in SDS (95% CI)^a^			
		Carotid intima-media thickness, mm (n = 3485)		Carotid distensibility, ×10^–3^ kPa^–1^ (n = 3316)	
		Confounder model	BMI model	Confounder model	BMI model
Fetal growth deceleration					
Infant growth deceleration	122	−0.02 (−0.20 to 0.17)	0.00 (−0.18 to 0.19)	0.12 (−0.08 to 0.32)	0.06 (−0.13 to 0.26)
Infant growth normal	382	−0.06 (−0.18 to 0.06)	−0.05 (−0.17 to 0.07)	0.02 (−0.11 to 0.14)	−0.01 (−0.13 to 0.12)
Infant growth acceleration	397	0.05 (−0.07 to 0.17)	0.03 (−0.09 to 0.15)	−0.08 (−0.20 to 0.05)	−0.04 (−0.16 to 0.09)
Fetal growth normal					
Infant growth deceleration	336	−0.00 (−0.13 to 0.12)	0.01 (−0.11 to 0.14)	0.10 (−0.03 to 0.23)	0.06 (−0.07 to 0.18)
Infant growth normal	812	[Reference]	[Reference]	[Reference]	[Reference]
Infant growth acceleration	400	0.19 (0.07 to 0.31)^b^	0.17 (0.05 to 0.28)^b^	−0.16 (−0.28 to −0.03)^b^	−0.09 (−0.21 to 0.04)
Fetal growth acceleration					
Infant growth deceleration	422	−0.00 (−0.12 to 0.11)	−0.00 (−0.12 to 0.12)	−0.03 (−0.15 to 0.09)	−0.03 (−0.15 to 0.09)
Infant growth normal	469	0.12 (0.01 to 0.23)^b^	0.11 (−0.004 to 0.22)	−0.04 (−0.16 to 0.07)	−0.01 (−0.13 to 0.10)
Infant growth acceleration	145	0.10 (−0.08 to 0.27)	0.07 (−0.11 to 0.24)	−0.09 (−0.27 to 0.09)	−0.01 (−0.18 to 0.17)

Abbreviations: BMI, body mass index (calculated as weight in kilograms divided by height in meters squared); SDS, standard deviation score.

^a^ Values are regression coefficients (95% CIs) and reflect the differences in carotid intima-media thickness (SDS) and carotid distensibility (SDS) for fetal and infant growth patterns from multivariable linear regression models. Estimates are from multiple imputed data. The confounder model is adjusted for child age at the outcome visit and sex, maternal age, prepregnancy BMI, educational level, race and ethnicity, folic acid use, smoking, and gestational hypertensive disorders. The BMI model is the confounder model additionally adjusted for sex-adjusted and gestational age–adjusted child BMI at the outcome measurement.

^b^ *P* < .05.

## Discussion

In this population-based prospective cohort study, we observed that higher fetal and infant weight growth patterns were associated with increased cIMT and lower carotid distensibility. Children with normal fetal growth that was followed by accelerated infant growth had the highest cIMT and lowest carotid distensibility. Childhood BMI seems to be involved in the pathways underlying the observed associations and was largely explanatory.

We observed that birth weight was positively associated with cIMT and negatively associated with carotid distensibility. Large size for gestational age was associated with increased cIMT and lower carotid distensibility, whereas SGA was associated with decreased cIMT and higher carotid distensibility. Gestational age at birth and low birth weight were not associated with cIMT or carotid distensibility. Our findings do not suggest associations of low birth weight and preterm birth with impaired arterial health. This finding is in contrast to previous studies.^29,30,31^ This difference in findings might be due to our relatively healthy population of children not born very preterm or with very low birth weight. Alternatively, the association of SGA and preterm birth with increased cIMT might be stronger among infants than among older children.^10^ Regarding the association of higher birth weight with increased cIMT and decreased carotid distensibility, a study in California of 670 children aged 11 years reported that higher birth weight was associated with increased cIMT, whereas no association was found for lower birth weight.^32^ A study of 696 participants in Finland reported that adults who were born at term and LGA had increased cIMT and higher risk of obesity at 24 to 45 years of age.^12^ A study of 2281 adults aged 24 to 45 years reported increased cIMT in children born preterm or with restricted fetal growth.^33^ In a British retrospective cohort study of 181 people approximately 70 years of age, lower birth weight was associated with a higher risk of carotid atherosclerosis.^18^ In contrast to these studies, no associations of low birth weight or SGA with the development of adverse arterial health were observed in our study. Because of the changes in prevalence of underweight, overweight, and obesity in the last decades, the associations might differ between birth cohorts. Previous studies have suggested nonlinear associations of birth weight and cIMT.^10,12^ However, we did not detect nonlinear associations in our study. Further research is needed to identify optimal ranges of weight ranges for fetal and infant growth. Overall, our results suggest that, among contemporary children, a higher birth weight followed by a higher childhood BMI is associated with an increased risk of adverse arterial health.

Because fetal and infant weight growth are strongly correlated, it is important to study the associations of fetal and infant weight combined. Higher fetal weight, birth weight, and infant weight at 6, 12, and 24 months were associated with increased cIMT independently from prior weights. Infant weight at 6, 12, and 24 months was also associated with decreased carotid distensibility. To our knowledge, our study is the first that assessed the independent critical periods for weight development from fetal life to infancy and the association with markers of arterial health. Both fetal life and infancy seem to be critical.

Normal fetal growth that was followed by accelerated infant growth was associated with the highest childhood cIMT even after adjustment for childhood BMI. However, an Australian cohort study of 140 children at 14 years of age showed no association of BMI trajectory in early life with cIMT.^34^ In line with our findings, a Brazilian prospective cohort study of 5914 participants at 30 years of age reported that weight gain in the first 2 years of life was positively associated with cIMT.^15^ Similarly, results from an Australian prospective cohort study of 395 children at 8 years of age showed that excessive weight gain between birth and 18 months was positively associated with cIMT.^16^ Thus, body weight changes in the first 2 years of life seem to be critical in the development of atherosclerotic changes of the carotid arteries.

Higher birth weight and weight gain in childhood is associated with obesity later in life.^4,35,36,37,38,39,40^ Obesity is a major risk factor in the development of atherosclerosis and arterial stiffness and, subsequently, cardiovascular disease.^39,40^ Weight gain in infancy and early childhood is associated with the risk of developing obesity, high blood pressure, and cardiovascular disease and its precursors in adulthood.^14,35,36,37,38,41^ Our effect estimates were largely explained by childhood BMI, suggesting that childhood BMI is involved in the pathways underlying the observed associations.

Well-known risk factors for cardiovascular disease, such as BMI, blood pressure, and lipid concentrations, are known to track from childhood into adulthood.^42,43,44^ It is not well known to what extent arterial health markers track from childhood to adulthood. Previous studies have shown associations of higher BMI, blood pressure, and plasma lipid concentrations among children with increased cIMT and arterial stiffness among adults.^45,46,47,48^ Among adults, an increase of 0.1 mm of cIMT is associated with myocardial infarction and stroke.^49^ The results of our study suggest that weight growth during fetal life and infancy is associated with arterial health in adulthood. Therefore, optimizing growth in early life, and especially preventing childhood obesity, might be beneficial for arterial health in childhood and adulthood. Whether measurements of arterial health in childhood add to prevention strategies to improve cardiovascular health throughout the life course should be further studied.

### Strengths and Limitations

This study has some strengths, including the population-based prospective cohort study design, large number of participants, detailed data on weight measurements from birth to 2 years of age, and information on carotid atherosclerotic markers at an early age.

This study also has some limitations. Of the 8631 singleton live births with information on fetal or infant growth, 4484 had data on childhood carotid measurements. Mothers of children not included in the analyses were more often nulliparous, more often of non-European ethnicity, more often smoked, and used folic acid supplements less often. Children not included were slightly more often born preterm and had a lower estimated fetal weight in the third trimester of pregnancy. These differences may be associated with the generalizability of our results, for our population seems to have slightly more favorable characteristics. Although we demonstrated high reproducibility in measuring the cIMT and carotid distensibility, we cannot completely rule out observer bias. Additionally, we adjusted for a large number of potential confounders, but residual confounding might still be a possibility owing to the observational nature of the study.

## Conclusions

This cohort study suggests that higher fetal and infant weight growth are associated with early markers of impaired arterial health in children at 10 years of age. Childhood BMI seems to be involved in the underlying pathways of the observed associations. Future studies are needed to assess potential causal pathways and to study how these pathways are associated with the development of early atherosclerotic changes in adulthood.

## Notes

Supplement.
eMethods.
**eTable 1.** Observed Participant Characteristics of the Study Population (N = 4484) **eTable 2.** Nonresponse Analysis in Singleton Live Births With and Without Outcome Measurements **eTable 3.** Associations of Birth Outcomes With Childhood Carotid Measurements, Basic Models **eTable 4.** Associations of Fetal and Infant Growth With Childhood Carotid Measurements From Conditional Analyses, Basic Models **eTable 5.** Associations of Fetal and Infant Weight Measurements With Childhood Carotid Measurements, Basic Models **eTable 6.** Associations of Fetal and Infant Growth Patterns With Childhood Carotid Measurements, Basic Models **eTable 7.** Maternal Prepregnancy BMI for Fetal and Infant Growth Patterns (n = 3485) **eTable 8.** Sensitivity Analysis Associations of Fetal and Infant Growth Patterns With Childhood Carotid Measurements **eFigure 1.** Flow Chart of the Study Population **eFigure 2.** Directed Acyclic Graph (DAG) Depicting the Associations Between Fetal and Infant Weight Measurements, Carotid Intima-Media Thickness, and Distensibility at 10 Years and Potential Covariates, Confounders, and Mediators
eReference.
