# Supplementary material for: Associations of Fetal and Infant Growth Patterns With Early Markers of Arterial Health in School-Aged Children
Source: JAMA Netw Open. 2022 Jun 29;5(6):e2219225. doi: 10.1001/jamanetworkopen.2022.19225 (PMC9244605; doi:10.1001/jamanetworkopen.2022.19225)
Supplement: Supplement. — eMethods. eTable 1. Observed Participant Characteristics of the Study Population (N = 4484) eTable 2. Nonresponse Analysis in Singleton Live Births With and Without Outcome Measurements eTable 3. Associations of Birth Outcomes With Childhood Carotid Measurements, Basic Models eTable 4. Associations of Fetal and Infant Growth With Childhood Carotid Measurements From Conditional Analyses, Basic Models eTable 5. Associations of Fetal and Infant Weight Measurements With Childhood Carotid Measurements, Basic Models eTable 6. Associations of Fetal and Infant Growth Patterns With Childhood Carotid Measurements, Basic Models eTable 7. Maternal Prepregnancy BMI for Fetal and Infant Growth Patterns (n = 3485) eTable 8. Sensitivity Analysis Associations of Fetal and Infant Growth Patterns With Childhood Carotid Measurements eFigure 1. Flow Chart of the Study Population eFigure 2. Directed Acyclic Graph (DAG) Depicting the Associations Between Fetal and Infant Weight Measurements, Carotid Intima-Media Thickness, and Distensibility at 10 Years and Potential Covariates, Confounders, and Mediators eReference. [file jamanetwopen-e2219225-s001.pdf]

Supplementary Online Content

Gonçalves R, Wiertsema CJ, Silva CCV, et al. Associations of fetal and infant growth patterns with early markers of arterial health in school-aged children. *JAMA Netw Open.* 2022;5(6):e2219225. doi:10.1001/jamanetworkopen.2022.19225

eMethods.
eTable 1. Observed Participant Characteristics of the Study Population (N = 4484)
eTable 2. Nonresponse Analysis in Singleton Live Births With and Without Outcome Measurements
eTable 3. Associations of Birth Outcomes With Childhood Carotid Measurements, Basic Models
eTable 4. Associations of Fetal and Infant Growth With Childhood Carotid Measurements From Conditional Analyses, Basic Models
eTable 5. Associations of Fetal and Infant Weight Measurements With Childhood Carotid Measurements, Basic Models
eTable 6. Associations of Fetal and Infant Growth Patterns With Childhood Carotid Measurements, Basic Models
eTable 7. Maternal Prepregnancy BMI for Fetal and Infant Growth Patterns (n = 3485)
eTable 8. Sensitivity Analysis Associations of Fetal and Infant Growth Patterns With Childhood Carotid Measurements
eFigure 1. Flow Chart of the Study Population
eFigure 2. Directed Acyclic Graph (DAG) Depicting the Associations Between Fetal and Infant Weight Measurements, Carotid Intima-Media Thickness, and Distensibility at 10 Years and Potential Covariates, Confounders, and Mediators
eReference.

This supplementary material has been provided by the authors to give readers additional information about their work.

© 2022 Gonçalves R et al. *JAMA Network Open.*

eMethods.

First trimester measures

We constructed gestational age adjusted standard deviation score (SDS) for first trimester fetal crown to rump length [1]. First trimester fetal growth was assessed in a subgroup of mothers who had a fetal first trimester crown to rump measurement within the range of 10 weeks 0 days to 13 weeks 6 days and had a reliable gestational age estimate based on the last menstrual period and a regular menstrual cycle (n = 941).

Childhood common carotid artery intima media thickness and distensibility

When children visited the research facility at the median age of 9.7 years (95% range, 9.3-10.5 years), we measured carotid intima media thickness (cIMT) and distensibility three times at both common carotid arteries using the Logiq E9 device (GE Medical Systems, Wauwatosa, WI, USA). Children were in the supine position, with the head tilted slightly away from the transducer. The common carotid artery was identified in a longitudinal plane, ~10 mm proximal from the carotid bifurcation. We obtained six recordings that ideally included multiple heart cycles. The analyses were performed offline and semi-automatically, using the application Carotid Studio (Cardiovascular Suite (Quipu srl, Pisa, Italy)). For each recording, at all R-waves of the simultaneous ECG, cIMT was computed at the 'far wall' as the average distance between lumen-intima and media-adventitia borders. The average cIMT of all frames of the acquired image sequence was computed. Distensibility was defined as the relative change in lumen area during systole for a given pressure change. Lumen diameter was computed as the average distance between the far and near media-adventitia interfaces, for each frame of the acquired image sequence. Distension was calculated as the difference between the maximal (diastolic) and minimal (systolic) lumen diameter. Per recording, the average distension and diameter values were used to compute average distensibility. Further data processing was performed in R. Children with at least one successful cIMT or

© 2022 Gonçalves R et al. *JAMA Network Open.*

distensibility measurement were included. The overall mean cIMT (mm) and distensibility ($kPa^{-1}*10^{-3}$) were used as main outcomes of interest. For the final analyses, distensibility was log-transformed to deal with a skewed distribution. We constructed standard deviation score values ((observed value−mean)/SD) for the childhood outcome measures to enable comparison of effect estimates. In a reproducibility study performed among 47 subjects, the interobserver and intraobserver intraclass correlation coefficient were greater than 0.85.

© 2022 Gonçalves R et al. *JAMA Network Open.*

eTable 1. Observed Participant Characteristics of the Study Population (N = 4484)[a]

Characteristics	Number of available cases	Participants, No. (%)
**Maternal**		
Age at enrolment, median (95% range), y	4484	31.2 (20.3, 39.6)
Pre-pregnancy BMI, median (95% range), kg/m^2	3749	22.6 (18.1, 34.6)
Nulliparous	4459	2613 (58.6)
Education level, higher education	4258	2134 (50.1)
Race and ethnicity	4401	
Cape Verdean	182	182 (4.1)
Dutch	2530	2530 (57.5)
Dutch Antilles	95	95 (2.2)
Moroccan	209	209 (4.7)
Surinamese	338	338 (7.7)
Turkish	312	312 (7.1)
Other[b]	735	735 (16.7)
Continued smoking during pregnancy	4015	961 (23.9)
Did not use folic acid supplement	3444	752 (21.8)
**Fetal**		
First trimester		
Gestational age, median (95% range), wk	941	12.4 (10.6, 13.8)
Crown rump length, mean (SD), mm	941	61.0 (11.6)
Second trimester		
Gestational age, median (95% range), wk	4295	20.5 (18.6, 23.3)
Estimated fetal weight, median (95% range), g	4238	364 (246, 624)
Third trimester		
Gestational age, median (95% range), wk	4379	30.4 (28.5, 33.0)
Estimated fetal weight, median (95% range), g	4352	1605 (1179, 2218)
**Birth**		
Child sex, female	4484	2260 (50.4)
Gestational age at birth, median (95% range), wk	4484	40.1 (35.9, 42.3)
< 37	207	207 (4.6)
37-42	4060	4060 (90.5)
>42	217	217 (4.8)
Birth weight, median (95% range), g	4479	3450 (2255, 4485)
< 2500	196	196 (4.4)
2500-4500	4182	4182 (93.4)
> 4500	101	101 (2.3)
Sex- and gestational age adjusted birth weight	4476	
Small (<10th percentile)	447	447 (10.0)
Appropriate (10th-90th percentile)	3582	3582 (80.0)
Large (>90th percentile)	447	447 (10.0)
**Infant**		

© 2022 Gonçalves R et al. *JAMA Network Open.*

At 6-mo visit		
Age at visit, median (95% range), mo	3401	6.2 (5.2, 8.3)
Weight, median (95% range), kg	3385	7.8 (6.2, 9.8)
At 12-mo visit		
Age at visit, median (95% range), y	3120	11.1 (10.1, 12.5)
Weight, median (95% range), kg	3103	9.6 (7.7, 11.8)
At 24-mo visit		
Age at visit, median (95% range), y	2937	24.8 (23.4, 28.2)
Weight, median (95% range), kg	2930	12.8 (10.3, 16.2)
**Childhood**		
Age at follow-up, median (95% range), y	4484	9.7 (9.3, 10.5)
BMI median (95% range), kg/m^2	4469	17.0 (14.0, 24.9)
Carotid intima media thickness, mean (SD), mm	4484	0.46 (0.04)
Carotid distensibility, median (95% range), kPaˆ-1*10ˆ-3	4304	55.9 (37.1, 85.5)

BMI: body mass index, mm: millimeter, kg: kilograms. Values are mean (SD), median (95% range), or number (valid %). [a:] Characteristics are based on observed not imputed data. [b:] Included ethnicities other than specified in table: African, American non-western, American western, Asian non-western, Asian western, European, Indonesian and Oceanian.

© 2022 Gonçalves R et al. *JAMA Network Open.*

eTable 2. Nonresponse Analysis in Singleton Live Births With and Without Outcome Measurements

**Characteristics**	**Children included in the analysis** (n = 4484)	**Children not included in the analysis** (n = 4141)*	**p-value**
**Maternal**			
Age at enrolment, median (95% range), y	31.2 (20.3, 39.6)	28.8 (18.5, 38.7)	<0.001
Pre-pregnancy BMI, median (95% range), kg/m^2	22.7 (17.8, 34.1)	22.7 (17.7, 35.5)	0.06
Parity, n nulliparous (%)	2628 (58.6)	2177 (52.5)	<0.001
Education level, n higher education (%)	2187 (48.8)	1287 (31.1)	<0.001
Ethnicity, n European (%	2908 (64.9)	1936 (46.8)	<0.001
Smoking during pregnancy, n continued (%)	1081 (24.1)	1273 (30.7)	<0.001
Folic acid supplement use, n did not use (%)	1072 (23.9)	1637 (39.5)	<0.001
**Fetal**			
First trimester			
Gestational age, median (95% range), weeks	12.4 (10.6, 13.8)	12.4 (10.8, 13.9)	0.97
Crown rump length, mean (SD), mm	61.0 (11.6)	60.8 (11.1)	0.76
Second trimester			
Gestational age, median (95% range), weeks	20.5 (18.6, 23.3)	20.5 (18.5, 23.8)	0.40
Estimated fetal weight, median (95% range), grams	364 (246, 624)	364 (240, 656)	0.81
Third trimester			
Gestational age, median (95% range), weeks	30.4 (28.5, 33.0)	30.3 (28.0, 33.0)	0.01
Estimated fetal weight, median (95% range), grams	1605 (1179, 2218)	1580 (1141, 2200)	<0.001
**Birth**			
Child sex, n female (%)	2260 (50.4)	2015 (48.7)	0.10
Gestational age at birth, median (95% range), weeks	40.1 (35.9, 42.3)	40.0 (35.3, 42.3)	0.02
< 37 weeks, n (%)	207 (4.6)	250 (6.0)	0.01
37-42 weeks, n (%)	4060 (90.5)	3678 (88.9)	0.01
>42 weeks, n (%)	217 (4.8)	210 (5.1)	0.01
Birth weight, median (95% range), grams	3450 (2255, 4485)	3400 (2210, 4490)	<0.001
< 2500 grams, n (%)	196 (4.4)	219 (5.4)	0.10
2500-4500 grams, n (%)	4182 (93.4)	3766 (92.3)	0.10
> 4500 grams, n (%)	101 (2.3)	95 (2.3)	0.10
Sex- and gestational age adjusted birth weight			
Small (<10th percentile), n (%)	447 (10.0)	455 (11.2)	0.18
Appropriate (10th-90th percentile), n (%)	3582 (80.0)	3228 (79.3)	0.18
Large (>90th percentile), n (%)	447 (10.0)	388 (9.5)	0.18
**Infant**			
At 6 months visit			
Age at visit, median (95% range), months	6.2 (5.2, 8.3)	6.2 (5.2, 8.3)	0.45
Weight, median (95% range), kg	7.8 (6.2, 9.8)	7.9 (6.2, 9.9)	0.00
At 12 months visit			
Age at visit, median (95% range), years	11.1 (10.1, 12.5)	11.1 (10.1, 12.6)	0.38
Weight, median (95% range), kg	9.6 (7.7, 11.8)	9.7 (7.7, 12.1)	0.00

© 2022 Gonçalves R et al. *JAMA Network Open.*

At 24 months visit			
Age at visit, median (95% range), years	24.8 (23.4, 28.2)	24.8 (23.4, 28.2)	0.21
Weight, median (95% range), kg	12.8 (10.3, 16.2)	12.9 (10.2, 16.3)	0.17

BMI: body mass index, mm: millimeter, kg: kilograms. * Children who did not participate in the 10-year follow-up or did not have childhood carotid measurements or had cardiac abnormalities for which exclusion was indicated. Values are mean (SD), median (95% range), or number (valid %). Differences in subject characteristics between the groups were evaluated using Independent Student T-test and Mann-Whitney U for continuous variables and χ^2 tests for categorical variables.

© 2022 Gonçalves R et al. *JAMA Network Open.*

eTable 3. Associations of Birth Outcomes With Childhood Carotid Measurements, Basic Models

	Difference in standard deviation score (95% CI)	
	**Carotid intima media thickness** (n = 4484)	**Carotid distensibility** (n = 4304)
**Birth outcomes**	Basic Model	Basic Model
**Birth weight**		
< 2500 grams (n = 196)	-0.04 (-0.18, 0.10)	0.10 (-0.05, 0.25)
2500-4500 grams (n = 4182)	*Reference*	*Reference*
> 4500 grams (n = 101)	0.13 (-0.06, 0.33)	-0.24 (-0.44, -0.04)*
Continuously (per 500 grams)	0.06 (0.04, 0.09)*	-0.05 (-0.08, -0.03)*
**Size for gestational age at birth**		
Small <10th percentile (n = 447)	-0.12 (-0.22, -0.02)*	0.12 (0.02, 0.22)*
Appropriate 10th-90th percentile (n = 3582)	*Reference*	*Reference*
Large >90th percentile (n = 447)	0.09 (-0.01, 0.18)	-0.11 (-0.21, -0.01)*
Continuously (per 1 SD grams)	0.07 (0.04, 0.09)*	-0.07 (-0.10, -0.04)*
**Gestational age at birth**		
< 37 weeks (n = 207)	-0.08 (-0.22, 0.06)	0.04 (-0.10, 0.18)
37-42 weeks (n = 4060)	*Reference*	*Reference*
>42 weeks (n = 217)	0.06 (-0.07, 0.20)	0.02 (-0.12, 0.16)
Continuously (per week)	0.02 (-0.001, 0.03)	-0.00 (-0.02, 0.01)

CI: Confidence interval. **P* value <0.05. Values are regression coefficients (95% confidence interval) that were obtained from multivariable linear regression models and reflect the differences in carotid intima media thickness (SDS) and carotid distensibility (SDS) for birth outcomes. Estimates are from multiple imputed data. The basic model is adjusted for child age at the outcome visit and sex.

© 2022 Gonçalves R et al. *JAMA Network Open.*

eTable 4. Associations of Fetal and Infant Growth With Childhood Carotid Measurements From Conditional Analyses, Basic Models

	**Difference in standard deviation score (95% CI)**	
	**Carotid intima media thickness** (n = 2249)	**Carotid distensibility** (n = 2137)
**Infant and fetal weight standard deviation score**	Basic model	Basic model
At 20 weeks	0.03 (-0.02, 0.07)	-0.01 (-0.06, 0.03)
At 30 weeks	0.07 (0.03, 0.11)**	-0.04 (-0.08, 0.01)
At birth	0.04 (0.004, 0.08)*	-0.01 (-0.05, 0.04)
At 6 months	0.05 (0.01, 0.09)*	-0.05 (-0.09, -0.01)*
At 12 months	0.05 (0.01, 0.09)*	-0.06 (-0.10, -0.01)*
At 24 months	0.06 (0.02, 0.10)**	-0.11 (-0.15, -0.06)**

CI: Confidence interval. **P* value <0.05. ***P* value <0.001. Values are regression coefficients (95% confidence interval) and reflect the differences in carotid intima media thickness (SDS) and carotid distensibility (SDS) per SDS change in infant and fetal weight from conditional models. Conditional regression analysis take into account the correlations between the weight measurement to identify independent critical weight periods. Standardized residuals resulting from linear regression models of weight regressed on all prior weights were used to construct weight variables, statistically independent from weight at earlier time points. Estimates are from multiple imputed data. The basic model is adjusted for child age at the outcome visit and sex.

© 2022 Gonçalves R et al. *JAMA Network Open.*

eTable 5. Associations of Fetal and Infant Weight Measurements With Childhood Carotid Measurements, Basic Models

Infant and fetal weight standard deviation score	Difference in standard deviation score (95% CI)					
	Carotid intima media thickness (n = 4484)			Carotid distensibility (n = 4304)		
	Basic model	Confounder model	BMI model	Basic model	Confounder model	BMI model
At first trimester (n = 942)	-0.01 (-0.07, 0.06)	-0.00 (-0.07, 0.06)	-0.00 (-0.07, 0.07)	-0.03 (-0.10, 0.04)	-0.03 (-0.10, 0.04)	-0.03 (-0.10, 0.03)
At 20 wk (n = 4238)	0.02 (-0.01, 0.05)	0.03 (-0.01, 0.06)	0.02 (-0.01, 0.05)	-0.03 (-0.06, -0.001)*	-0.03 (-0.06, 0.001)	-0.03 (-0.06, 0.01)
At 30 wk (n = 4352)	0.05 (0.03, 0.08)*	0.06 (0.03, 0.09)*	0.06 (0.03, 0.09)*	-0.05 (-0.08, -0.02)*	-0.05 (-0.08, -0.02)*	-0.04 (-0.07, -0.01)*
At birth (n = 4476)	0.07 (0.04, 0.09)*	0.08 (0.05, 0.11)*	0.07 (0.04, 0.10)*	-0.07 (-0.10, -0.04)*	-0.07 (-0.10, -0.04)*	-0.05 (-0.08, -0.02)*
At 6 mo (n = 3385)	0.10 (0.07, 0.14)*	0.11 (0.07, 0.14)*	0.09 (0.06, 0.13)*	-0.07 (-0.11, -0.04)*	-0.07 (-0.10, -0.03)*	-0.02 (-0.06, 0.02)
At 12 mo (n = 3103)	0.12 (0.08, 0.15)*	0.13 (0.09, 0.17)*	0.12 (0.08, 0.16)*	-0.11 (-0.15, -0.07)*	-0.10 (-0.14, -0.06)*	-0.05 (-0.09, -0.01)*
At 24 mo (n = 2930)	0.13 (0.09, 0.16)*	0.14 (0.10, 0.18)*	0.13 (0.09, 0.17)*	-0.16 (-0.19, -0.12)*	-0.14 (-0.19, -0.11)*	-0.10 (-0.14, -0.08)*

CI: Confidence interval. **P* value <0.05. Values are regression coefficients (95% confidence interval) that were obtained from multivariable linear regression models and reflect the differences in carotid intima media thickness (SDS) and carotid distensibility (SDS) for birth outcomes. Estimates are from multiple imputed data. The basic model is adjusted for child age at the outcome visit and sex. The confounder model is the basic model additionally adjusted for maternal age, pre-pregnancy body mass index, educational level, ethnicity, folic acid use, smoking and gestational hypertensive disorders. The BMI model is the confounder model additionally adjusted for sex-and gestational age adjusted child body mass index at the outcome measurement.

© 2022 Gonçalves R et al. *JAMA Network Open.*

eTable 6. Associations of Fetal and Infant Growth Patterns With Childhood Carotid Measurements, Basic Models

	**Difference in standard deviation score (95% CI)**	
	**Carotid intima media thickness** (n = 3485)	**Carotid distensibility** (n = 3316)
**Fetal and infant growth patterns**	Basic model	Basic model
Fetal growth deceleration		
Infant growth deceleration (n = 122)	-0.00 (-0.19, 0.18)	0.12 (-0.08, 0.32)
Infant normal growth (n = 382)	-0.06 (-0.18, 0.06)	0.01 (-0.11, 0.14)
Infant growth acceleration (n = 397)	0.05 (-0.07, 0.17)	-0.09 (-0.22, 0.03)
Fetal normal growth		
Infant growth deceleration(n = 336)	0.00 (-0.12, 0.13)	0.10 (-0.03, 0.23)
Infant normal growth (n = 812)	*Reference*	*Reference*
Infant growth acceleration (n = 400)	0.19 (0.07, 0.31)*	-0.17 (-0.29, -0.05)*
Fetal growth acceleration		
Infant growth deceleration (n = 122)	-0.01 (-0.12, 0.11)	-0.04 (-0.16, 0.08)
Infant normal growth (n = 383)	0.11 (0.004, 0.22)*	-0.06 (-0.17, 0.06)
Infant growth acceleration (n = 397)	0.10 (-0.07, 0.27)	-0.11 (-0.28, 0.07)

CI: confidence interval.**P* value <0.05. Values are regression coefficients (95% confidence interval) and reflect the differences in carotid intima media thickness (SDS) and carotid distensibility (SDS) for fetal and infant growth patterns from multivariable linear regression models. Estimates are from multiple imputed data. The basic model is adjusted for child age at the outcome visit and sex.

© 2022 Gonçalves R et al. *JAMA Network Open.*

eTable 7. Maternal Prepregnancy BMI for Fetal and Infant Growth Patterns (n = 3485)[a]

**Fetal and infant growth patterns**	**Pre-pregnancy BMI, median (95% range), kg/m^2**
Fetal growth deceleration	
Infant growth deceleration (n = 122)	22.6 (17.7, 34.0)
Infant normal growth (n = 382)	22.5 (17.9, 34.3)
Infant growth acceleration (n = 397)	22.3 (17.6, 36.7)
Fetal normal growth	
Infant growth deceleration (n = 336)	22.7 (17.8, 32.2)
Infant normal growth (n = 812)	22.2 (18.0, 33.9)
Infant growth acceleration (n = 400)	22.5 (18.0, 34.5)
Fetal growth acceleration	
Infant growth deceleration (n = 422)	22.7 (18.5, 35.8)
Infant normal growth (n = 469)	22.8 (18.4, 34.6)
Infant growth acceleration (n = 145)	23.4 (18.6, 36.3)

BMI: body mass index, m^2: square meter, kg: kilograms. Values are median (95% range).

[a:] Characteristics are based on the pooled results after multiple imputations.

© 2022 Gonçalves R et al. *JAMA Network Open.*

eTable 8. Sensitivity Analysis Associations of Fetal and Infant Growth Patterns With Childhood Carotid Measurements

	Difference in standard deviation score (95% CI)			
	**Carotid intima media thickness** (n = 2789)		**Carotid distensibility** (n = 2635)	
**Fetal and infant growth patterns**	Confounder model	BMI model	Confounder model	BMI model
Fetal growth deceleration				
Infant growth deceleration (n = 106)	-0.06 (-0.27, 0.14)	-0.04 (-0.24, 0.16)	0.11 (-0.11, 0.32)	0.04 (-0.17, 0.26)
Infant normal growth (n = 296)	-0.08 (-0.22, 0.05)	-0.07 (-0.21, 0.06)	-0.02 (-0.17, 0.12)	-0.05 (-0.19, 0.09)
Infant growth acceleration (n = 324)	0.02 (-0.11, 0.10)	0.01 (-0.13, 0.14)	-0.05 (-0.19, 0.08)	-0.01 (-0.15, 0.13)
Fetal normal growth				
Infant growth deceleration (n = 282)	0.02 (-0.12, 0.16)	0.04 (-0.10, 0.17)	0.12 (-0.02, 0.27)	0.08 (-0.06, 0.22)
Infant normal growth (n = 644)	*Reference*	*Reference*	*Reference*	*Reference*
Infant growth acceleration (n = 302)	0.27 (0.13, 0.40)**	0.24 (0.10, 0.37)**	-0.18 (-0.32, -0.04)*	-0.10 (-0.24, 0.04)
Fetal growth acceleration				
Infant growth deceleration (n = 377)	-0.01 (-0.14, 0.12)	-0.01 (-0.14, 0.12)	-0.00 (-0.14, 0.13)	-0.00 (-0.14, 0.13)
Infant normal growth (n = 385)	0.14 (0.01, 0.26)*	0.13 (0.004, 0.25)*	-0.06 (-0.19, 0.07)	-0.03 (-0.16, 0.10)
Infant growth acceleration (n = 113)	0.08 (-0.11, 0.28)	0.05 (-0.14, 0.25)	-0.06 (-0.26, 0.14)	0.01 (-0.19, 0.22)

CI: Confidence interval, BMI: body mass index. **P* value <0.05.

Sensitvity analysis including only children with weight at 24 months old. Values are regression coefficients (95% confidence interval) and reflect the differences in carotid intima media thickness (SDS) and carotid distensibility (SDS) for fetal and infant growth patterns from multivariable linear regression models. Estimates are from multiple imputed data. The confounder model is adjusted for child age at the outcome visit and sex, maternal age, pre-pregnancy body mass index, educational level, ethnicity, folic acid use, smoking and gestational hypertensive disorders. The BMI model is the confounder model additionally adjusted for sex-and gestational age adjusted child body mass index at the outcome measurement.

© 2022 Gonçalves R et al. *JAMA Network Open.*

eFigure 1. Flow Chart of the Study Population

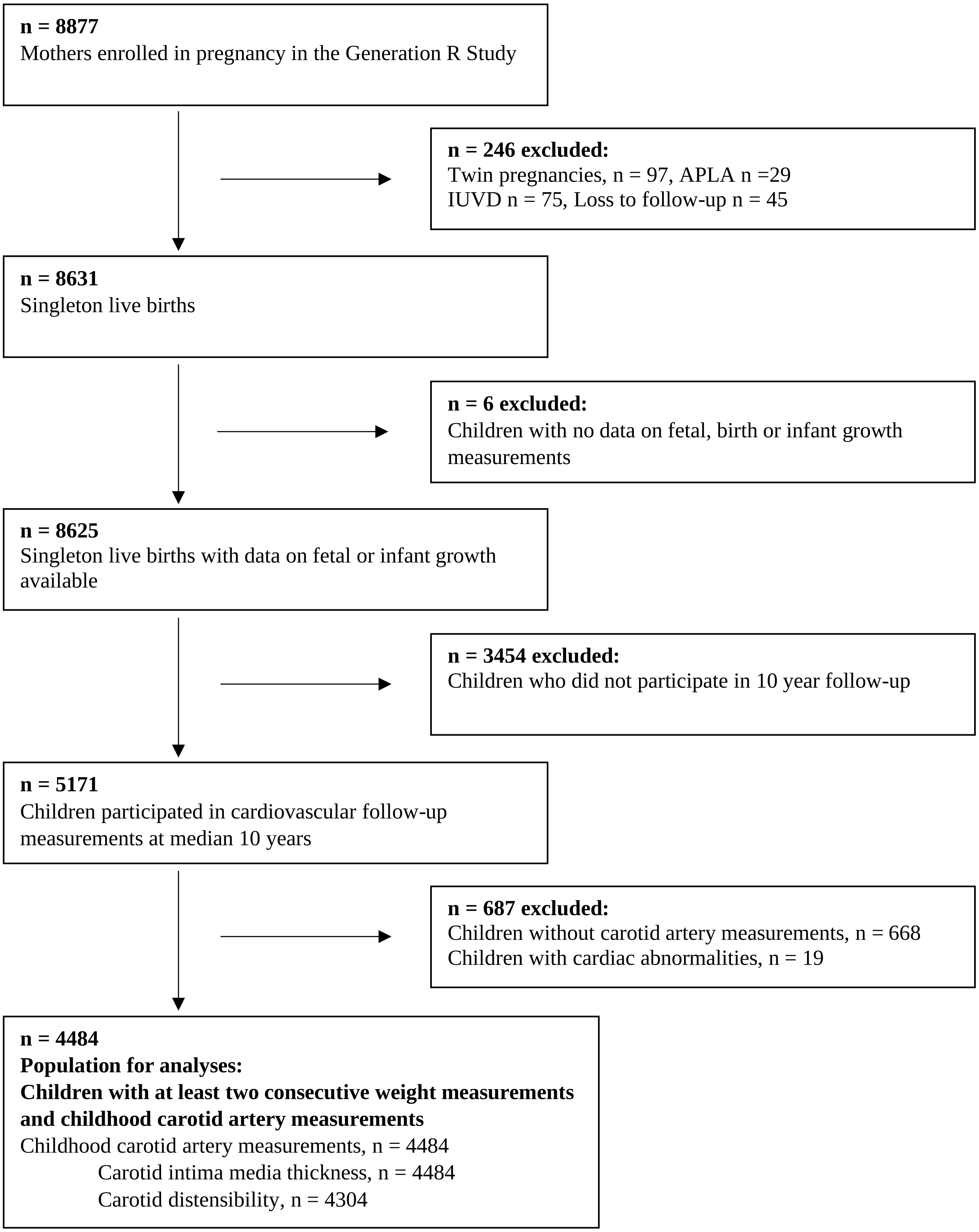

© 2022 Gonçalves R et al. *JAMA Network Open.*

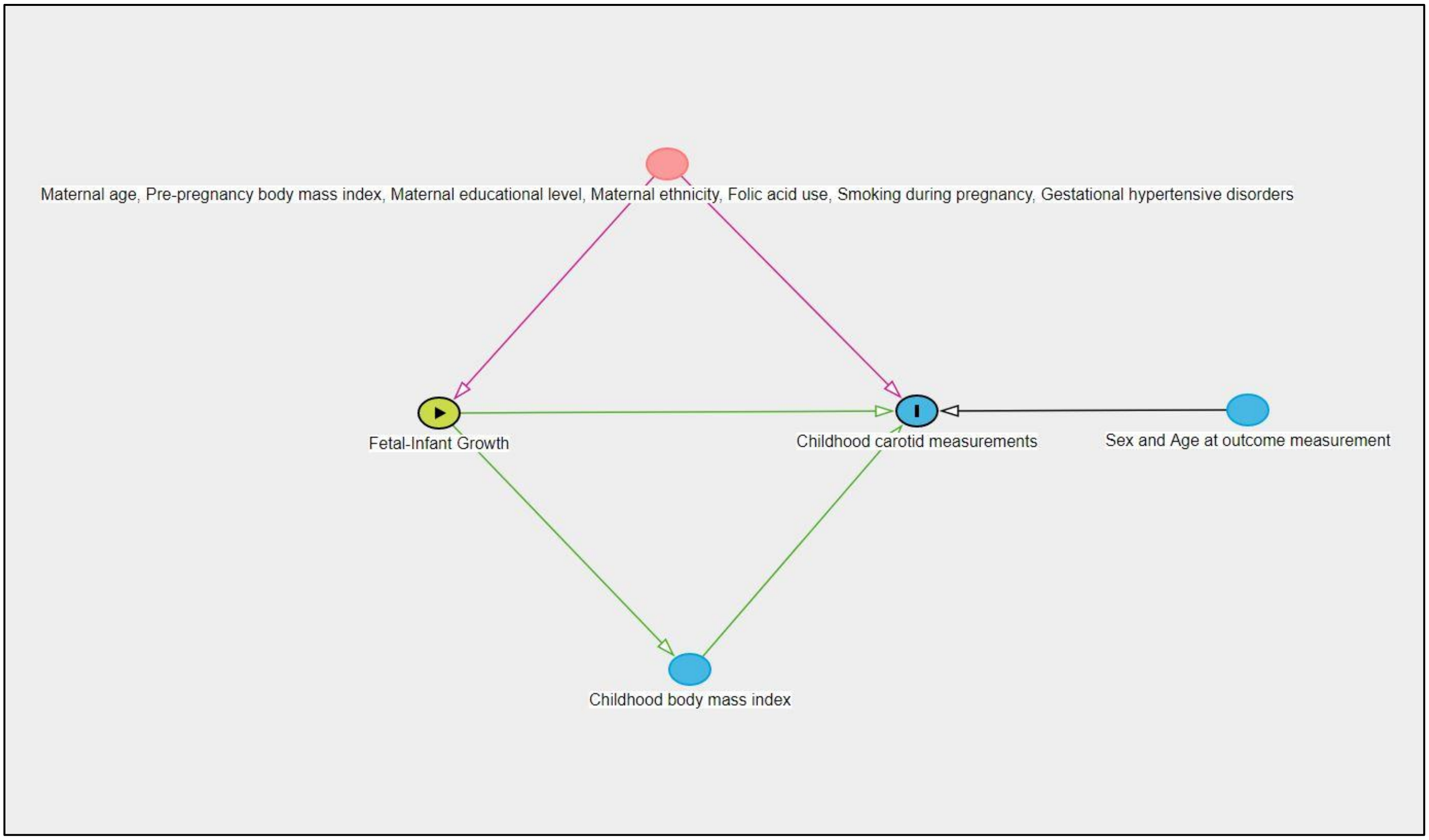

eFigure 2. Directed Acyclic Graph (DAG) Depicting the Associations Between Fetal and Infant Weight Measurements, Carotid Intima-Media Thickness, and Distensibility at 10 Years and Potential Covariates, Confounders, and Mediators

© 2022 Gonçalves R et al. *JAMA Network Open.*

eReference.

1. Mook-Kanamori, D.O., et al., *Risk factors and outcomes associated with first-trimester fetal growth restriction.* JAMA, 2010. **303**(6): p. 527-34.

© 2022 Gonçalves R et al. *JAMA Network Open*.
